# A Giant Nabothian Cyst Associated With Secondary Infertility: A Case Report

**DOI:** 10.7759/cureus.115606

**Published:** 2026-09-01

**Authors:** Yassine Belhaj, Kenza Benchaaboune, Sofia Jayi, Fatima Zahra Fdili Alaoui, Hekmat Chaara, Moulay Abdelillah Melhouf

**Affiliations:** 1 Obstetrics and Gynaecology 2, Centre Hospitalier Universitaire Hassan II, Fes, MAR

**Keywords:** case report, cervical cyst, cervical factor infertility, conservative management, giant nabothian cyst, secondary infertility

## Abstract

Nabothian cysts are common benign retention cysts of the uterine cervix and are usually small, asymptomatic, and incidentally detected. Giant nabothian cysts are rare and may become clinically significant because of mass effect, cervical distortion, symptoms, diagnostic uncertainty, or possible interference with fertility in selected patients. We report the case of a 32-year-old woman with secondary infertility for two years and no significant medical, surgical, or gynecologic history. Before referral, she had not undergone a complete infertility evaluation or detailed per-speculum cervical examination and had waited for spontaneous conception. At our department, speculum examination revealed a large translucent and tense cystic mass occupying almost the entire vaginal cavity and arising from the anterior cervical lip. Pelvic ultrasonography showed a well-circumscribed unilocular anechoic cervical cyst measuring 53 x 50 mm, with regular contours and no solid or complex components. A standard infertility work-up performed after referral was otherwise unremarkable, including ovulation and hormonal assessment, tubal patency evaluation, pelvic ultrasound, and partner semen analysis. The patient was not receiving ovulation induction or hormonal infertility treatment at the time of drainage. Conservative puncture under local anesthesia aspirated 100 mL of viscous mucoid fluid, and a biopsy of the cyst wall was performed. Cytologic examination showed benign endocervical-type epithelial elements without atypia or malignant cells. Histopathologic examination showed mucin-filled cystic spaces lined by benign columnar, cuboidal, or flattened epithelial cells, without cytologic atypia, notable mitotic activity, dysplasia, or malignancy, confirming a nabothian cyst. Clinical and ultrasound follow-up showed resolution of the cyst without recurrence. The patient conceived spontaneously two months after treatment, and the pregnancy resulted in a live birth according to available follow-up. This case highlights a temporal association between conservative treatment of a giant nabothian cyst and subsequent conception, without proving direct causality. Large cervical masses require careful clinical examination, appropriate imaging, broad differential diagnosis including cystic and non-cystic lesions, and cytologic or histopathologic confirmation when diagnostic uncertainty exists.

## Introduction

Nabothian cysts, also known as mucinous retention cysts of the cervix, are benign lesions resulting from obstruction of endocervical glands and accumulation of mucus. They are frequently observed in women of reproductive age and are usually identified incidentally during routine gynecologic examination or pelvic imaging [1,2]. Most are small and clinically insignificant. By contrast, unusually large or giant nabothian cysts are rarely reported, and their true incidence remains poorly established [1-3].

Most nabothian cysts are asymptomatic. When large, they may present with pelvic pressure, dyspareunia, vaginal fullness, a protruding cervical or vaginal mass, abnormal vaginal discharge, abnormal bleeding, dysmenorrhea, cervical distortion, or obstructive symptoms depending on their size and location [1,4-6]. Reported complications of large cysts include mass effect, diagnostic confusion with other cervical lesions, urinary or vaginal obstructive symptoms in selected cases, and rarely labor obstruction or prolapse-like presentations [4-6].

The differential diagnosis of a large cervical or vaginal mass includes both cystic and non-cystic lesions. Cystic lesions include giant nabothian cysts, Gartner duct cysts, vaginal inclusion cysts, cervical endometriotic cysts, tunnel clusters, and other multicystic glandular cervical lesions. Non-cystic lesions, such as cervical polyps, cervical ectropion or hypertrophy, and submucosal leiomyomas, may present with a similar clinical appearance. Importantly, large or multicystic cervical lesions should be distinguished from malignant or premalignant glandular lesions, particularly adenoma malignum, especially when imaging shows irregular margins, multiloculation, solid components, deep stromal extension, watery discharge, or abnormal bleeding [2,7,8].

Diagnosis is usually based on clinical examination and imaging findings, especially ultrasound. Magnetic resonance imaging (MRI) may be helpful when ultrasound findings are inconclusive or when the lesion is large, complex, multiloculated, or suspicious for malignant disease [2,7,8]. Cytologic and histopathologic evaluation may be necessary when the lesion is unusually large, atypical, symptomatic, or diagnostically uncertain. Benign findings typically include absence of cytologic atypia or malignant cells and a cyst wall lined by benign endocervical-type mucinous epithelium, without dysplasia or malignancy.

The relationship between giant nabothian cysts and infertility remains uncertain. Large cysts may theoretically reduce fertility by distorting the external cervical canal, altering cervical mucus drainage, or partially interfering with sperm passage. Majeed et al. reported a cross-sectional sonographic study of infertile women and suggested a significant association between the number and size of nabothian cysts and infertility [9]. However, observational data do not establish causality. Documented cases of spontaneous conception following treatment of a giant nabothian cyst remain extremely limited [3]. We report a case of a giant nabothian cyst associated with secondary infertility and spontaneous pregnancy after conservative treatment, while emphasizing that this temporal association does not establish direct causality.

## Case presentation

A 32-year-old woman was referred to our department for evaluation of secondary infertility that had persisted for two years. She had no significant medical, surgical, or gynecologic history. Her obstetric history included two previous pregnancies and vaginal deliveries. Before presentation to our department, the patient had not undergone a complete specialized infertility evaluation or detailed gynecologic examination, including per-speculum assessment of the cervix. She had mainly waited for spontaneous conception for two years before seeking specialized care.

During the two-year period of secondary infertility, the patient reported regular menstrual cycles. She denied amenorrhea, cyclic pelvic pain, hematocolpos, abnormal uterine bleeding, abnormal vaginal discharge, dyspareunia, or other symptoms suggestive of complete genital tract obstruction. After referral to our department, a standard infertility work-up was performed and was unremarkable. Ovulatory function and hormonal assessment were normal, tubal patency was confirmed, pelvic ultrasound showed no abnormality other than the cervical cyst, and the partner's semen analysis was within normal limits. No other female or male infertility factor was identified. The patient was not receiving ovulation induction or hormonal infertility treatment at the time of cyst drainage.

At our department, per-speculum examination immediately revealed a large translucent and tense cystic mass occupying almost the entire vaginal cavity and apparently arising from the anterior lip of the cervix (Figure 1). Vaginal examination confirmed a renitent mass related to the cervix. The lesion was considered to cause distortion and possible partial mechanical obstruction of the cervical canal rather than complete obstruction.

**Figure 1:**
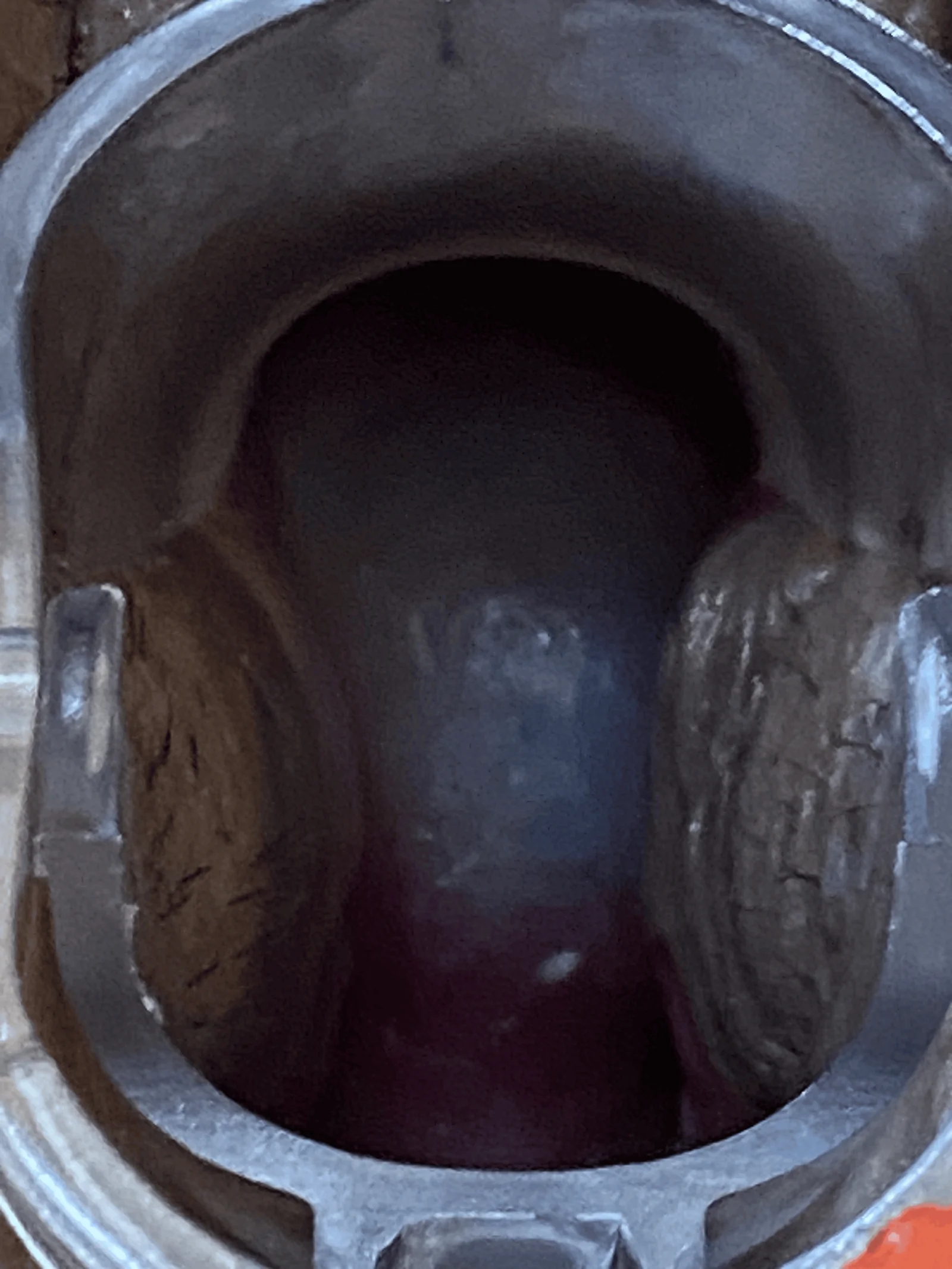
Speculum examination showing a large translucent cervical cystic mass protruding into and almost filling the vaginal cavity

The initial clinical differential diagnosis included both cystic and non-cystic lesions, including giant nabothian cyst, Gartner duct cyst, vaginal inclusion cyst, cervical endometriotic cyst, tunnel clusters, cervical polyp, cervical ectropion or hypertrophy, prolapsed cervical or submucosal leiomyoma, and malignant glandular cervical lesions such as adenoma malignum. The translucent and tense appearance of the mass and its continuity with the anterior cervical lip favored a benign cervical cyst, but confirmatory evaluation was required because of the unusually large size. Pelvic ultrasonography demonstrated a well-circumscribed unilocular anechoic cyst measuring 53 x 50 mm, with regular contours and a thin wall, in continuity with the anterior lip of the cervix (Figure 2). No internal solid component, papillary projection, septation, irregular wall thickening, or other complex feature was identified. The remainder of the pelvic ultrasound examination was unremarkable.

**Figure 2:**
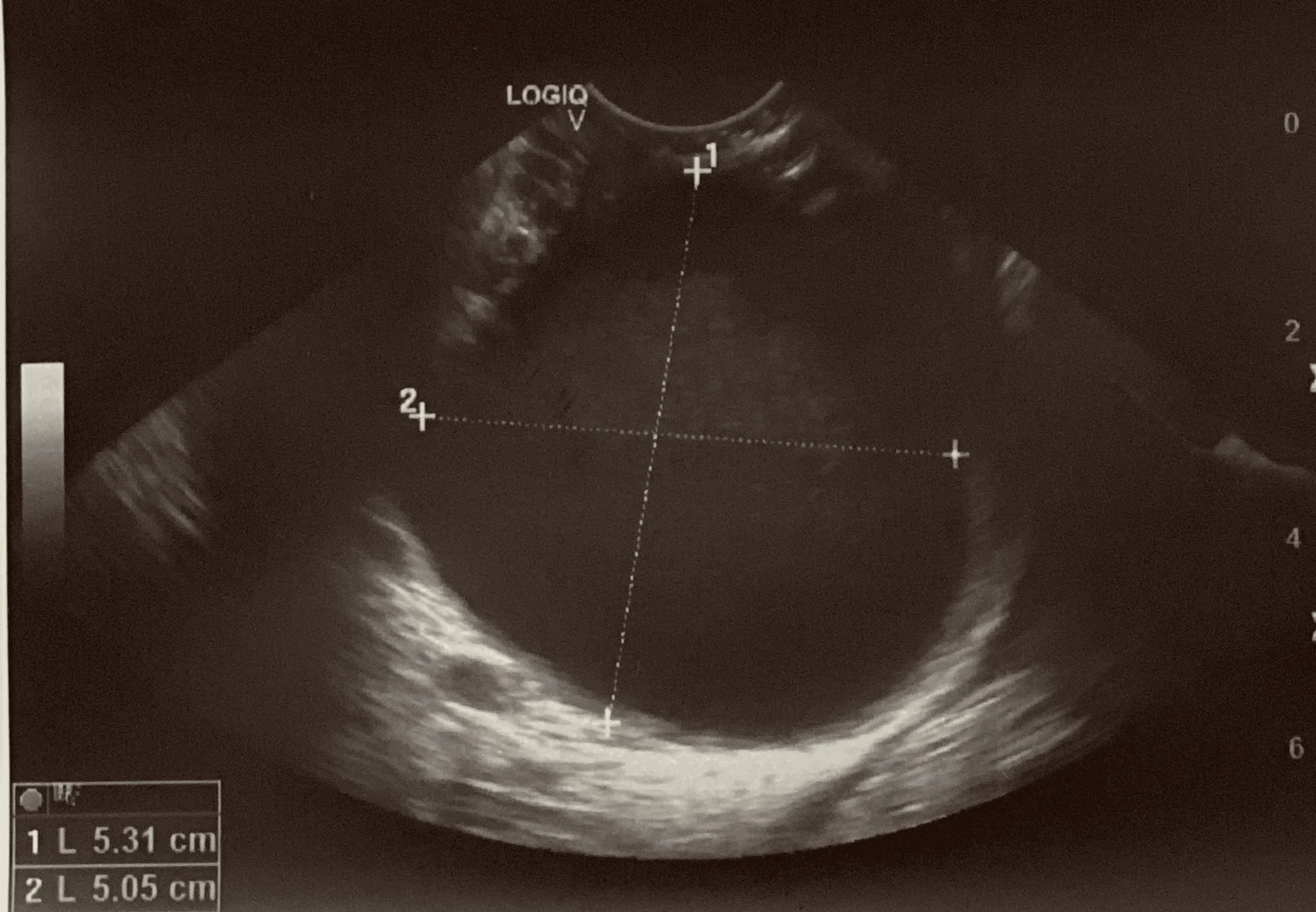
Pelvic ultrasound showing a well-defined anechoic cervical cyst measuring approximately 53 x 50 mm, continuous with the anterior lip of the cervix

MRI was not performed because the ultrasound findings were considered reassuring, showing a well-circumscribed, unilocular, anechoic cyst with regular contours and no suspicious solid or complex components. In addition, clinical judgment and local resource considerations supported initial conservative management. Diagnostic confirmation was subsequently obtained through cytologic examination of the aspirated fluid and histopathologic examination of the cyst wall.

After counseling, a conservative diagnostic and therapeutic approach was chosen. Under local anesthesia, puncture of the cyst aspirated 100 mL of viscous mucoid fluid, which was sent for cytologic analysis. The reported aspirated volume was based on the procedural measurement at the time of drainage and not on photographic estimation. A biopsy of the cyst wall was also performed for histopathologic examination.

Cytologic examination of the aspirated mucoid fluid showed a mucoid background with benign endocervical-type epithelial elements, without cytologic atypia or malignant cells. Histopathologic examination of the cyst wall showed mucin-filled cystic spaces lined by benign columnar, cuboidal, or flattened epithelial cells, without cytologic atypia or notable mitotic activity. No dysplasia or malignancy was identified. These findings were consistent with a benign nabothian cyst.

After drainage, clinical follow-up showed resolution of the cyst, with no symptoms or clinical evidence of cyst recurrence. Ultrasound follow-up also confirmed resolution of the cyst. The patient conceived spontaneously two months after the procedure. According to available follow-up at the time of revision, the pregnancy had resulted in a live birth.

## Discussion

This case illustrates an uncommon presentation of a giant nabothian cyst in a woman with secondary infertility. Although nabothian cysts are generally incidental and clinically harmless, large lesions may become symptomatic because of mass effect, cervical distortion, or diagnostic uncertainty. In the present case, the lesion was not an incidental imaging finding; it was clinically detected on speculum examination as a large translucent cervical mass. This reinforces the importance of careful gynecologic examination, including per-speculum evaluation of the cervix, in women presenting with infertility.

Large cervical masses require a broad differential diagnosis before treatment. Common cystic lesions include giant nabothian cysts, Gartner duct cysts, vaginal inclusion cysts, cervical endometriotic cysts, tunnel clusters, and other multicystic glandular cervical lesions. Non-cystic lesions that may present with a similar clinical appearance include cervical polyps, cervical ectropion or hypertrophy, and submucosal leiomyomas. Malignant or premalignant cervical glandular lesions, particularly adenoma malignum, should be considered when the lesion is large, multiloculated, irregular, associated with solid components, abnormal bleeding, watery discharge, or suspicious imaging features [2,7,8].

In this patient, the clinical and ultrasound findings favored a benign cyst: the lesion was translucent and tense on speculum examination, continuous with the anterior cervical lip, well circumscribed, unilocular, anechoic, and regular on ultrasound, without solid or complex components. MRI was not performed because these findings were reassuring and because clinical judgment and resource availability supported initial conservative management. Nevertheless, given the large size of the lesion and the need to exclude atypia or malignancy, cytologic analysis of the aspirated fluid and histopathologic examination of the cyst wall were performed, both confirming benign disease.

The association between giant nabothian cysts and infertility remains uncertain. In the present case, the standard infertility evaluation was otherwise unremarkable, including ovulatory and hormonal assessment, tubal patency, pelvic ultrasound except for the cervical cyst, and partner semen analysis. The patient was not receiving ovulation induction or hormonal infertility treatment at the time of drainage. She also reported regular menstruation and no symptoms of complete genital tract obstruction. Therefore, the lesion should not be described as causing complete cervical obstruction. A more cautious interpretation is that its large size and location may have distorted normal cervical anatomy and possibly caused partial mechanical obstruction of the cervical canal, potentially interfering with sperm passage in this selected case.

The available literature linking nabothian cysts to infertility is limited. Turan et al. reported spontaneous pregnancy after treatment of an obstructive nabothian cyst [3]. Majeed et al. evaluated the sonographic association between nabothian cysts and infertility in a cross-sectional analytical study including infertile women aged 18-45 years who underwent transvaginal ultrasound [9]. The authors reported nabothian cysts in women with primary and secondary infertility and suggested a significant correlation between the number and size of nabothian cysts and infertility [9]. However, because this study was observational, it supports an association rather than a direct causal relationship. Our case adds to this limited evidence by describing a large clinically detected cervical nabothian cyst associated with secondary infertility, followed by spontaneous conception after conservative treatment.

The occurrence of spontaneous pregnancy two months after cyst aspiration may appear surprising, but it remains biologically plausible because conception may occur within the first cycles after restoration of cervical anatomy. Nevertheless, this timing represents a temporal association and should not be interpreted as definitive evidence of a direct causal relationship. Given the absence of other identified infertility factors and the subsequent restoration of cervical anatomy, the giant nabothian cyst may have represented a contributing and potentially reversible cervical factor. This interpretation remains limited by the single-patient nature of the report.

Management of nabothian cysts depends on symptoms, size, diagnostic certainty, and reproductive goals. Small asymptomatic cysts generally require no treatment. Symptomatic, enlarging, obstructive, atypical, or diagnostically uncertain lesions may require drainage, excision, marsupialization, or biopsy. In women desiring fertility preservation, conservative management may be appropriate when imaging is reassuring and malignancy has been reasonably excluded. Histopathological confirmation remains important when diagnostic uncertainty exists, especially in large or atypical cervical cystic lesions.

This report has limitations. A single case cannot establish a causal relationship between treatment of the cyst and pregnancy. Further reports are needed to better define the relationship between giant nabothian cysts and fertility outcomes.

## Conclusions

Giant nabothian cysts are rare benign cervical lesions that may become clinically significant when they distort the cervix or partially obstruct the cervical or vaginal canal. In selected women with infertility and an otherwise unremarkable infertility work-up, a large cervical cyst distorting the cervical canal may be considered as a potential contributing cervical factor, although causality cannot be established from a single case report. This case highlights a temporal association between conservative treatment of a giant nabothian cyst and subsequent spontaneous conception, followed by a favorable pregnancy outcome according to available follow-up. Careful clinical examination, appropriate imaging, broad differential diagnosis including cystic and non-cystic lesions, and cytologic or histopathologic confirmation when diagnostic uncertainty exists are essential. Conservative management may restore normal cervical anatomy and may potentially improve reproductive outcomes in carefully selected patients, particularly when fertility preservation is desired.
